# Challenges in the Management of and Biologic Use in Incarcerated Patients With Inflammatory Bowel Disease

**DOI:** 10.1093/crocol/otad002

**Published:** 2023-02-02

**Authors:** Sarah Barbina, John Romano, Erin Forster

**Affiliations:** Department of Internal Medicine, Medical University of South Carolina, Charleston, SC, USA; Division of Gastroenterology and Hepatology, Department of Internal Medicine, Medical University of South Carolina, Charleston, SC, USA; Division of Gastroenterology and Hepatology, Department of Internal Medicine, Medical University of South Carolina, Charleston, SC, USA

**Keywords:** inflammatory bowel disease, incarceration, vulnerable, prisoner, biologic therapy

## Abstract

**Background:**

Therapy and management of inflammatory bowel disease (IBD) require commitment from both the provider and patient to ensure optimal disease management. Prior studies show vulnerable patient populations with chronic medical conditions and compromised access to health care, such as incarcerated patients, suffer as a result. After an extensive literature review, there are no studies outlining the unique challenges associated with managing prisoners with IBD.

**Methods:**

A detailed retrospective chart review of 3 incarcerated patients cared for at a tertiary referral center with an integrated patient-centered IBD medical home (PCMH) and a review of literature was performed.

**Results:**

All 3 patients were African American males in their 30s with severe disease phenotypes requiring biologic therapy. All patients had challenges with medication adherence and missed appointments related to inconsistent access to clinic. Two of the 3 cases depicted better patient-reported outcomes through frequent engagement with the PCMH.

**Conclusions:**

It is evident there are care gaps and opportunities to optimize care delivery for this vulnerable population. It is important to further study optimal care delivery techniques such as medication selection, though interstate variation in correctional services poses challenges. Efforts can be made to focus on regular and reliable access to medical care, especially for those who are chronically ill.

## Introduction

Inflammatory bowel disease (IBD) is a relapsing and remitting inflammatory condition describing ulcerative colitis and Crohn’s disease (CD). The incidence is rising with 6.8 million people worldwide living with IBD.^1^ Therapies for moderate severity IBD involve either injection or infusion biologic therapy, though oral options are more available. Shared decision-making (SDM) is at the center of drug selection. However, the use of biologics requires frequent healthcare contact for optimal outcomes, a challenging reality for vulnerable populations such as those currently incarcerated.^2^

The highest age-standardized prevalence rate of IBD worldwide is in North America, a continent that includes the United States, which is known for the highest incarceration rate worldwide and the largest number of prisoners. Fortunately, our therapeutic options for the treatment of IBD are also increasing. SDM discussions between patient and provider are at the center of drug selection, and final choices are often influenced by route of administration and frequency of dosing. Vulnerable populations, such as those currently incarcerated, may require special considerations to ensure optimal outcomes.

In review of the literature, there are ***no*** studies outlining the unique challenges associated with managing IBD in these patients. We aim to describe the clinical course of several incarcerated patients at our tertiary referral center and the obstacles impacting their clinical course. This study describes 3 specific patients and the evolution of their SDM and the efficacy of their disease management.

## Methods

A retrospective case series with data extraction from electronic medical records from December 2019 to present and literature review was performed.

### Case 1

A 30-year-old incarcerated African American (AA) male with a history of an abdominal gunshot wound was admitted from jail with diarrhea and hypovolemia. Computed tomography (CT) showed terminal ileal inflammation. Colonoscopy was consistent with Crohn’s ileitis. Ustekinumab (UST) was selected as initial therapy and infused on the day of discharge. This choice was based on leveraging the weight-based initial dose and ability to ensure adherence, with confirmation of future dosing through nurse-led injection appointments, and consistent access to medication with patient assistance programs. Unfortunately, due to transportation issues, he had multiple missed doses with a gap of as long as 6 months. However, he was reloaded with an IV dose, and with intensive social work support to coordinate logistics, he received one maintenance dose prior to being released from his detention center. Unfortunately, he remains lost to follow-up by both the clinic and specialty pharmacy, a challenge not uncommon in relation to the correctional system and recidivism. 

### Case 2

A 35-year-old incarcerated AA male was admitted to the hospital several times for hematochezia with multiple unrevealing endoscopic evaluations. He was readmitted for a gastroenterology (GI) bleed, and magnetic resonance enterography (MRE) and fecal calprotectin were again unremarkable. Repeat colonoscopy showed severe inflammation of the terminal ileum (TI) with patchy mild–moderate inflammation of colon and pathology consistent with idiopathic IBD. He was started on IV steroids and discharged on an oral prednisone taper for 9 weeks. Initial planned outpatient therapy was 5 mg/kg infliximab, but he experienced a delayed start requiring an additional steroid taper. Infliximab was started 4 months later at standard dosing. He unfortunately was late for his second dose and missed his third dose due to hospitalization for ongoing symptoms. During that admission, his anemia was so severe that a blood transfusion was required. At that time, the decision was made to reinduce with an increased dose at 10 mg/kg every 4 weeks with the first dose on the day of discharge. Since that time, he has been late for multiple infusions due to prison-related scheduling issues. After requiring his fifth prolonged prednisone taper in 1 year, he underwent infliximab reload at 10 mg/kg. He again was late for his every 4-week maintenance infusions. Since being released from prison, he has been on time for infliximab infusions 10 mg/kg every 8 weeks with reported improvement in his diarrhea and hematochezia. Subsequent endoscopy revealed resolution of the TI disease with patchy mild colitis in only 1 region of the colon. His dosing regimen was optimized to 10 mg/kg every 4 weeks and clinically he has been doing well.

### Case 3

A 31-year-old incarcerated AA male complained of hematochezia and fever requiring admission to the hospital and was diagnosed with *Clostridium difficile* colitis. CT scan and colonoscopy showed left-sided colitis. Following treatment with oral vancomycin, outpatient colonoscopy was consistent with residual proctosigmoiditis. Through SDM, he was started on mesalamine enemas but had difficulty retaining them and decision was made to start on UST for ease of dosing and avoidance of per-rectum therapies per patient preference. The UST was infused at the clinic during a scheduled visit. He missed multiple doses due to inconsistent transport to clinic for nurse-led administration of medication. He was then released from custody and off all therapy until developing *C. difficile* infection requiring hospitalization. He was treated with vancomycin and then resumed on PO and PR mesalamine as an outpatient. However, upon reincarceration with questionable access to medication, he developed worsening symptoms and was started on sulfasalazine. Repeat colonoscopy showed Mayo 3 pancolitis with pathology confirming moderate inflammation. He resumed UST therapy with a standard loading dose given at a clinic appointment and 90 mg SC every 8 weeks consistently while incarcerated. The patient has since been released from the detention center and has a steady job. He has been in frequent contact with the PCMH and the behavioral health social worker who assists him in coming to appointments and receiving his medication in a timely fashion from the specialty pharmacy. Clinically, he is doing well and is planned for endoscopic evaluation shortly once his insurance is valid. Additional biochemical evaluation is pending given the cost associated with self-pay laboratory studies.

## Discussion

All 3 patients were incarcerated AA males who experienced difficulty in obtaining routine care and consistent access to biologic therapy for IBD. Two out of the 3 patients were on primarily injection-based chronic therapies to best coincide with planned healthcare system encounters—either clinic or nurse visits. These visits can be coordinated with preidentified correctional facility personnel in advance but do require anonymity to avoid jailbreak attempts. Care delivered in the context of a medical home utilizes social workers/care coordinators to navigate these potential logistic challenges. It is evident there are care gaps as shown by loss to follow-up related to correctional transfers, recidivism, and missed doses of medication—even for those medications chosen for their relative dosing *infrequency* (every 8 weeks). Consideration should be given toward favoring IV “reload” doses over shortening dosing frequency to avoid gaps in administration. Two of the 3 cases clearly depict high healthcare utilization but do reflect that with the assistance of a medical home and frequent contact with correctional facilities, one can overcome barriers to ensure appropriate care for better clinical outcomes. It is important to further study the best way to deliver IBD care, specifically biologics, to this vulnerable population. 

Although there is *no* research or case reports on IBD management in incarcerated individuals, there is literature on difficulty of chronic disease management for other diagnoses: 60% of inmates with a diagnosis requiring routine lab work had never undergone a blood draw.^3^ One qualitative study stated that 1 patient with CD was unable to receive proper ileostomy bags and therefore suffered from leaking bags.^4^ Furthermore, a study showed only 38% of patients with diabetes were given their prescribed aspirin.^5^ Similarly, IBD patients require more than just consistent medication administration; standard of care requires regular lab monitoring, vaccine administration, and cancer screenings.^6^ As seen in all 3 patients, concomitant vitamin and mineral deficiencies are commonly requiring repletion. Weekly rather than daily Vitamin D supplementation could be utilized given less-demanding dosing regimens along with parenteral rather than oral formulations of iron for similar reasons. Here we suggest biologic medications well suited for infrequent and subcutaneous administration and utility of a PCMH for the coordination of care for incarcerated patients. Therapeutic drug monitoring can be challenging to coordinate but should be utilized at least reactively in times of clinical decompensation and could be performed during nurse-led injection visits as needed.

## Conclusion

For incarcerated patients, the successful administration of biologic therapy for maximal medical effectiveness requires operationalizing SDM. For those with compromised access to care, as is seen in the inmate population, it is of paramount importance to attempt to standardize and optimize care pathways. For example, choosing therapies with similar efficacy but less-demanding monitoring or administration schedules may be ideal for those in correctional facilities. As seen in this patient population, erratic access to care and medications, despite the selection of the least frequently administered, noninfusion-based therapies, compromised efficacy, and ultimately resulted in worse outcomes. Advocacy efforts with organizations supporting incarcerated individuals focusing on regular and reliable access to medical care are needed. SDM conversations are not limited to those patients who live a life beyond bars.

**Table 1.** Demographic information, disease type, current therapy, and difficulties of the 3 cases studied.

	Case 1	Case 2	Case 3
Age	38	35	31
Race	African American	African American	African American
Gender	Male	Male	Male
IBD type	Crohn’s disease	Crohn’s disease	Ulcerative colitis
Extraintestinal manifestations	Primary sclerosing cholangitis, arthralgias	None reported	None reported
Comorbidities	Gunshot wound with large bowel resection	Anemia, saddle pulmonary embolism	Recurrent clostridium difficile infection
Vitamin and mineral deficiencies	Iron, Vit D deficient	Vit D, B12, and Iron deficient	Iron, Vit D deficient
Current therapy	Ustekinumab 90 mg every 8 weeks	Infliximab 1000 mg (10 mg/kg) every 4 weeks	Ustekinumab 90 mg every 8 weeks
Missed therapy dose documented	Yes	Yes	Yes
Therapeutic drug monitoring performed	No	Yes	No
Drug level		13	
Antibodies present		No	
Hospital admissions since initial diagnosis	No	Yes	Yes

## Data Availability

Data not publicly available.
